# Effect of Increasing Dietary Aminoacid Concentration in Late Gestation on Body Condition and Reproductive Performance of Hyperprolific Sows

**DOI:** 10.3390/ani10010099

**Published:** 2020-01-08

**Authors:** Senén Seoane, Pasquale De Palo, José Manuel Lorenzo, Aristide Maggiolino, Pablo González, Leticia Pérez-Ciria, Maria Angeles Latorre

**Affiliations:** 1 Cooperativas Orensanas (COREN) Sociedad Cooperativa Galega, 32003 Ourense, Spain; senen.seoane@coren.es; 2 Department of Veterinary Medicine, University of Bari A. Moro, Italy, S.P. per Casamassima, km 3, Valenzano, 70010 Bari, Italy; pasquale.depalo@uniba.it (P.D.P.); aristide.maggiolino@uniba.it (A.M.); 3 Ctr. Tecnol. Carne Galicia, Rua Galicia 4, Parque Tecnol. Galicia, San Cibran De Vinas, 32900 Ourense, Spain; jmlorenzo@ceteca.net (J.M.L.); pablogonzalez@ceteca.net (P.G.); 4 IA2-Facultad de Veterinaria, Universidad de Zaragoza, Calle Miguel Servet 177, 50013 Zaragoza, Spain; leticiapcgm@gmail.com

**Keywords:** amino acid, pregnancy, backfat depth, performances, highly prolific dams

## Abstract

**Simple Summary:**

Nutrition during gestation is relevant for the success of reproductive sows and the late pregnancy is an especially critical period. Currently, the usual feeding management includes only one diet supplied at different levels through pregnancy and it could not be enough. Additionally, modern sows are producing large litters; their requirements are probably higher than those of commercial sows and they have to be met. With the current study, it can be concluded that a high level of amino acids in the diet provided approximately during the last month of gestation (around 10 g of standardized ileal digestibility lysine/kg of feed, with the remaining essential AA following the ideal protein concept) could be a strategy to improve the sow body condition and the reproductive performances.

**Abstract:**

A total of 62 highly prolific Danbred sows was used to evaluate the implications of increasing dietary amino acid (AA) concentration during late gestation (from day 77 to 107 of pregnancy) on body condition and reproductive performances. Sows were assigned to one of the two treatments (n = 31, with similar number of sows in the second-, third- and fourth-cycle); control diet (containing 6 g of standardized ileal digestible lysine -SID Lys-)/kg) and high AA level (containing 10 g SID Lys/kg and following the ideal protein concept for the remaining essential AA). On day 108 of pregnancy, animals were moved to the farrowing-lactating facilities where they spent until weaning receiving a common standard lactation diet. After farrowing, litters were standardized to 13 piglets each. At 107 d of gestation, backfat depth was thicker in sows fed high AA concentration than in those fed control diet (*p* < 0.0001) but these significant differences disappeared at weaning (*p* > 0.05). Additionally, at farrowing, the litter size (*p* = 0.043) and weight (*p* = 0.017) were higher in sows fed high AA level. It can be concluded that the increase in the AA content in the feed during the last month of gestation could improve the body condition of the sows and their performance results.

## 1. Introduction

The nutrient requirements in sows change through the pregnancy and therefore, the diet fed will have consequences on the dam metabolism and on the reproductive results. Some researchers have demonstrated that sow body condition and productive performances are not influenced by dietary energy concentration during pregnancy for several parities [1,2]. However, the great fetal development and growth of the mammary glands occur during late pregnancy and the protein (amino acids -AA) requirement is decisive [3]. Samuel et al. [4] concluded that Lys requirements increased substantially from early to late gestation suggesting that the phase feeding of pregnant sows should be considered to supply nutrients in accordance with nutrient demands. Most of the previous studies focused on the influence of adequate to extremely low lysine (Lys) intake, and only some of them evidenced the responses to relatively high Lys intake, being moderate the Lys increases tested (6 vs. 8 g total Lys/kg [5] or 4.6 vs. 7.4 g total Lys/kg [6]). It also has to be considered that excessive maternal fat gain during gestation should be avoided because it decreases voluntary feed intake during lactation [7].

Moreover, current highly prolific sows require especial nutritional strategies because of their larger body size and the considerable decline in body fat. The dietary needs for these new genetic lines have been researched in the last years and the recommendations should be revised [8,9].

Therefore, the aim of this trial was to determine the effects of increasing the dietary AA concentration during approximately the last month of gestation (increasing the standardized ileal digestible Lys -SID Lys- from 6 to 10 g/kg and maintaining the ideal protein concept for the remaining essential AA) on sow body condition and growth performance of hyperprolific multiparous sows.

## 2. Materials and Methods

### 2.1. Animal Husbandry and Experimental Design

The animals were cared for and managed during the study according to the European Community standards (Council Directive 2008/120/EC) [10]. The trial was conducted during a 10-month period (April–December 2016). A total of 62 hyperprolific Danbred sows from second-, third- and fourth-parity were used. All of them were in one contemporary group. Experimental animals were checked for oestrus two times per day (09:00 h and 15:30 h) using the back-pressure test in the presence of a mature teaser boar. Twenty-four hours after the first standing heat reflex, sows were inseminated with a commercial dose of semen (2 × 10^9^ sperm cells of a Pietrain boar line; Nucleus, France). If still in oestrus, dams received a second insemination 16 to 24 h after the first insemination. The sows were randomly assigned within parity to one of two treatments comprising a different dietary concentration of AA given from day 77 to 107 of gestation: 6 vs. 10 g SID Lys/kg of feed (there were a similar number of dams in the second-, third- and fourth-cycle in each group). The low level (6 g SID Lys/kg) was considered as the control considering that the estimated requirement of National Research Council (NRC) [11] for sows in second-, third- or fourth-parity from 90 days of gestation is between 5 and 6 g SID Lys/kg. The high level (10 g SID Lys/kg) was formulated to be above those recommendations and also in accordance with the findings reported in the literature [12]. A constant relationship was maintained of Lys with the remaining essential AA following the ideal protein concept [11].

### 2.2. Management and Controls

From day 1 to 107 of gestation, sows were housed in groups of 12. For feeding, animals were locked in the crates for 30 min to give each one the chance to eat its portion of the feed. Sows received 3 kg/d and 2.5 kg/d of a common gestation diet (2120 kcal net energy/kg and 6 g SID Lys/kg) from day 1 to 30 and from day 31 to 76 of gestation, respectively. From day 18 to 25 post-insemination, sows were checked for signs of oestrus twice daily using a mature teaser boar. If it was positive, the date of return to oestrus after the first insemination was recorded as the first date a standing heat reflex was observed. Around 4 weeks post-insemination, an ultrasound scan (WED-3000V, Well Medical Electronics Co., Ltd., Shenzhen, China) was performed to confirm pregnancy. It was decided that if an animal did not return to oestrus but was diagnosed as not pregnant, the date of the ultrasound scan would be recorded as the date when the sow was no longer pregnant.

From day 77 to 107 of gestation, the experimental diets were provided (Table 1) and the feeding level was increased to 3.0 kg/d. That feed was distributed twice per day (07:30 h and 14:30 h) and water was available ad libitum.

At day 108 of pregnancy (7 days prior to the estimated date for parturition), sows were moved to farrowing crates (2.6 × 1.8 m) which included a stall (2.1 × 0.5 × 0.9 m), where they stayed until weaning, receiving a commercial lactation diet. With the aim of avoiding feed wastage, the days before farrowing, the feed supply was gradually reduced to 2 kg on the day of farrowing (or lower, depending on sow appetite).

The feeding level during lactation was gradually increased from 2 kg/d to an ad libitum level until weaning. It was given as dry feed twice a day (07:30 h and 14:30 h) and the sows had free access to water via nipple drinkers placed in the feeder. Individual feed intake was recorded during the lactation period (from farrowing to weaning).

Individual backfat depth (BFD) and loin muscle depth (LMD) were measured at previous weaning, at days 77 and 107 of gestation and at weaning. The measures were taken using an ultrasound scan (WED-3000V, Well Medical Electronics Co., Ltd., Shenzhen, China), above the last rib on the left side at 6.0–6.5 cm from the midline.

At farrowing, piglets, including total born, alive and stillborn, were counted and individually weighed. At 24 h post-farrowing, litter size was equalized in a blinded manner to 13 piglets per litter and cross-fostering was minimized only being allowed among sows of the same dietary treatment. No pigs were added to litters thereafter. Removed pigs were put on an off-test nursing female. The number of pigs and their weights was recorded again after the litter standardization. Lactation lasted 28 ± 2 days as average and no creep feeding was provided to the piglets during that period. Healthy status in sows, piglet mortality and causes of death during lactation were checked and recorded. From day 2 post-weaning, sows were checked every morning in order to detect oestrus and were inseminated again. Weaning-to-mating interval was also recorded. By program design, sows that were not detected in oestrus after 7 days or more from weaning and also those that returned to oestrus after insemination are not maintained in the next cycle. The management during the subsequent cycle was the same as the previous one except for the feeding plan because all animals received the control diet (containing 6 g SID Lys/kg) from day 1 post-insemination to day 107 of gestation. Again, at farrowing, piglets, including total, alive and stillborn, were counted and individually weighed.

### 2.3. Statistical Analyses

Data were analyzed with the SAS package version 9.4 (SAS Institute, Cary, NC, USA). Firstly, the normality of the residuals of the variables studied was tested using the UNIVARIATE procedure. Sow body condition variables were analyzed using the MIXED procedure. The model included diet, time and their interaction as fixed effects and sows within treatment as the random effect. Time was analyzed with a repeated statement using Toeplitz covariance structure in the case of BFD and heterogeneous first-order autoregressive structure in the case of LMD, the models in each case with the smallest Schwarz Bayesian’s Information Criterion. Reproductive performance normal variables were analyzed using the GLM procedure. The model included the diet as the main effect. Reproductive performance variables whose residuals did not follow a normal distribution were analyzed with the Mann-Whitney U Test. The experimental unit was the sow in body condition variables and the litter in the case of reproductive performance variables. A *p*-value < 0.05 was considered a significant difference.

## 3. Results

The influence of the dietary AA level during the late gestation on BFD and LMD of highly prolific sows is shown in Table 2. No difference was observed among treatments in BFD or in LMD at the beginning of the trial (at previous weaning, *p* > 0.05), thus validating our randomization process. However, at 107 d of gestation, BFD was thicker in sows fed high AA concentration than in those fed the control diet (16.8 vs. 15.3 mm; *p* = 0.031). It was also observed when BFD gain was evaluated from day 77 to 107 of gestation (1.31 vs. 0.24 mm, respectively; *p* < 0.0001). However, these differences in BFD disappeared at weaning (*p* > 0.05). In fact, the BFD loss during lactation was higher by 27% in sows receiving more AA in the feed (*p* = 0.005). The length of gestation was similar in both groups (114.9 and 115.3 days for sows fed control- or high AA-diet, respectively).

In relation to LMD, although the dietary AA level did not have any effect at day 107 of gestation or at weaning (*p* > 0.05), the LMD loss during lactation was lower in the sows fed the highest AA content than in those fed the control diet (0.158 vs. 0.952 mm; *p* = 0.003).

When the period studied was longer (a full cycle; between two consecutive weanings), no influence was detected on BFD evolution (*p* > 0.05) but the diet including high AA concentration carried out a lower LMD loss (by 1.58 mm) than the control diet (*p* = 0.0002). The feed intake through lactation (from farrowing to weaning) was similar for both groups (*p* > 0.05).

The diet with high AA content improved some parameters related to productive performances in comparison with the control diet (Table 3).

At farrowing, it enhanced the number of live-born piglets (17.1 vs. 15.7; *p* = 0.043) and the litter weight (25.9 vs. 23.1 kg; *p* = 0.017). At weaning, it was detected that sows receiving high AA level in late gestation had heavier litters (73.0 vs. 67.1 kg; *p* = 0.053) and heavier piglets (5.79 vs. 5.40 kg; *p =* 0.034) than those receiving the control diet. Moreover, at subsequent parity, dams that had been fed previously high AA content had a higher total number of born piglets (*p* = 0.005) and also live-born piglets (*p* = 0.039).

## 4. Discussion

This study was designed to evaluate if a substantial increase of AA concentration in the diet provided during late gestation improves the body condition and the reproductive results in hyperprolific multiparous sows. The Lys is considered as the first limiting AA in typical corn-soybean meal diets and daily Lys intake is a primary factor determining dam performance [14]. In the current trial, the Lys levels tested were 6 vs. 10 g SID/kg with the remaining essential AA following the ideal protein concept.

According to the NRC [11], protein deposition and retention during gestation are divided into six tissue pools: fetus, placenta plus fluids, uterus, mammary tissue, time-dependent protein deposition, and energy-dependent protein deposition (time-dependent and energy-dependent pools are sow body pools). The Lys requirements for maintenance are low and almost constant, and the best opportunity for replenishing body protein reserves is in early gestation. Protein and AA demands for fetal growth and mammary development increase in an exponential manner in late gestation [15]. Kim et al. [16] described that a sow with 14 fetuses would need approximately 0.27 and 4.0 g SID Lys/day in early and late pregnancy, respectively, strictly for fetal growth, being similar for the requirement for mammary gland development (0.24 to 4.0 g SID Lys/day). Those authors [16] also indicated that the hyperprolific sows have a slightly higher Lys need than do normal sows based on simulations in which the records to work were 15 or 11.5 total born, respectively.

In a factorial approach [17], the authors quantified the energy and Lys requirements of sows during transition and lactation and also calculated the energy and Lys balances of sows during late gestation and lactation. These authors reported that almost all deposited energy and Lys in late gestation occur in the fetus, uterus, mammary and colostrum, whereas the retention in fat and muscle tissues regarding body condition happens in early-to mid-gestation. In the current trial, the increase in AA concentration from day 77 to 107 of pregnancy resulted in higher BFD at farrowing, which would confirm the results of other work [5] comparing diets including 6 or 8 g Lys/kg during the same period (from day 80 to 110 of gestation). Similar results were reported by Zhang et al. [6] who found that multiparous sows consuming 7.5 g Lys/kg during middle to late gestation (day 30 to 110) had higher BW gain and backfat thickness during pregnancy than sows consuming 4.5 g Lys/kg. Excessive dietary Lys levels must also be avoided because they cause a decrease in backfat thickness [18]. In a recent review [19], it was concluded that taking both energy and SID Lys requirements into account, it would be so difficult, almost impossible, to fulfill the requirements of all sows by using a single feed along all gestation.

During lactation, the high AA concentration provided during gestation could contribute to reduced loss in LMD indicating that muscle breakdown was higher in sows fed inadequate amounts of AA. Yang et al. [5] showed a higher creatinine level, which is an indicator of muscle catabolism, in sows fed low Lys diets at the day of post-farrowing and at weaning. When lactating sows are fed dietary AA levels below their needs, body protein tissue is catabolized in an attempt to supply the nutrients to maintain milk production. A sow can be catabolic in late pregnancy when the demands of the developing fetuses are greatest, but whether this happens and when, and the extent of the catabolic state depends on energy and nutrient supply [20].

The dietary AA concentration did not affect the number of pigs born and it could be explained that was because the experiment began after embryos were implanted [21]. However, the number of pigs that were live-born was higher with a higher concentration. Some works [22,23] found significant reductions in the probability of stillborns for gestating sows fed high Lys contents. It is interesting, as changes would be expected in the body composition (lean-to-fat ratio) of females fed a high AA intake, which could, in turn, positively impact uterine muscle tone and reduce dystocia [24].

The litter weight at farrowing increased as the AA level increased in the gestation diet. It is related to the higher litter size detected in those dams as no significant difference was detected in the piglet weight between treatments. However, certain unanimity can be found in the literature about the poor productive performances of litters nursed by sows fed lower Lys diets [25,26,27].

Weaning is known to be associated with dramatic metabolic and hormonal changes. Some authors detected that when Lys intake decreased, serum insulin concentration, FSH and LH are reduced [5] and yields of colostrum components are penalized [28]. Milk composition seems to be more affected by lactating feeding being related to a lower Lys intake with a lower concentration of dry matter and crude protein [29], although this is not clear [30]. However, it also has to be noted that the mammary protein content increased rapidly in the last 45 days of gestation [15] and feeding during late gestation could have an effect on it. Taking in the results of the current study account, we consider that the high dietary AA concentration in late gestation could contribute to produce heavier litters and piglets at weaning, although several factors have to be considered and it has also to be noted that litters were standardized the day post-farrowing. In this sense, although the mortality rate during lactation was not significant, our results (better with a high AA level) agree with those of Gonçalves et al. [23] who reported that pre-weaning mortality improved for litters suckling from females fed high amino acid intake compared with litters suckling from females fed low AA intake.

Perhaps one of the greatest findings in this study was that high AA content in the late gestation feed could contribute, at least in part, to increase the litter size in the subsequent cycle. In fact, sows fed the high AA level had 1.5 more live-born piglets than those fed the control diet. It suggests evidence for a possible medium-term impact or carryover effect of dietary treatments on reproductive results of sows.

## 5. Conclusions 

In conclusion, the increase in dietary AA concentration during approximately the last month of pregnancy (from 6 to 10 g SID Lys/kg, maintaining the ideal protein concept with the remaining essential AA) improves sow body condition. It is also adequate to guarantee good reproductive performances in the immediate parity and maybe even for the subsequent parity, although more research is needed to confirm this. Therefore, to meet AA requirements in late gestation, a phase feeding program with two diets is recommended with higher Lys levels during late gestation.

## Figures and Tables

**Table 1:** Composition of the diets tested from day 77 to 107 of gestation in hyperprolific multiparous sows (as-fed basis).

Item	Control	High Aminoacid Concentration
**INGREDIENTS (g/kg)**		
Barley	150.0	210.0
Wheat	199.4	248.5
Maize	124.7	20.5
Sugarcane molasses	5.0	0
Rapeseed meal 00	0	60.0
Animal blended fat	5.0	5.0
Sunflower meal 36% crude protein	65.5	0
Palm kernel meal	80.0	0
Soybean meal 47% crude protein	10.0	74.0
Maize DDGS	66.5	91.2
Wheat bran	191.0	185.0
Beet molasses	70.0	70.0
Soybean hulls	5.0	4.4
Calcium carbonate	13.2	12.0
Monocalcium phosphate	3.00	1.68
L-Lysine 50%	4.85	9.0
Threonine	0.84	2.72
Vitamin and mineral premix	2.00	2.00
Others (additives) ^2^	4.01	4.01
**NUTRIENTS (g/kg, Unless Otherwise Indicated) ^3^**		
Net energy (kcal/kg)	2119	2119
Dry matter	881.5	879.2
Total ash	53.1	51.6
Crude protein	137.9	165.0
Ether extract	36.0	30.0
Neutral detergent fiber	252.7	220.0
Starch	325.4	320.5
SID Lysine	0.60	1.00
SID Methionine	0.20	0.41
SID Methionine + Cystine	0.40	0.67
SID Threonine	0.43	0.72
SID Tryptophan	0.12	0.18

^1^ Standardized ileal digestible.^2^ Choline, phytases, antioxidants, mycotoxin sequestrant. ^3^ Calculated composition according to FEDNA [13].

**Table 2:** Effect of dietary amino acid (AA) concentration from day 77 to 107 of gestation on backfat depth and loin muscle depth in hyperprolific multiparous sows.

Variable	Diets ^1^		SE ^2^	*p*-Value
	Control	High AA		
**BACKFAT DEPTH (mm)**				
At previous weaning	12.87	12.82	0.496	0.945
At day 77 of gestation	15.04	15.52	0.496	0.504
At day 107 of gestation	15.29	16.83	0.496	0.031
At weaning	10.68	10.98	0.496	0.673
∆ from 77d to 107d of gestation	0.24	1.31	0.176	<0.0001
∆ from day 107 of gestation to weaning	−4.61	−5.85	0.303	0.005
∆ between two consecutive weaning	−2.19	−1.85	0.378	0.521
**LOIN MUSCLE DEPTH (mm)**				
At previous weaning	48.69	48.74	0.800	0.966
At day 77 of gestation	47.40	48.13	0.774	0.508
At day 107 of gestation	46.81	47.65	0.774	0.446
At weaning	45.86	47.49	0.792	0.150
∆ from 77d to 107d of gestation	−0.59	−0.48	0.095	0.416
∆ from day 107 of gestation to weaning	−0.95	−0.16	0.182	0.003
∆ between two consecutive weaning	−2.83	−1.25	0.287	0.0002
Feed intake through lactation (kg)	124.4	122.1	2.047	0.435

^1^ Control diet: 6 g standardized ileal digestible lysine -SID Lys-/kg; High AA diet: 10 g SID Lys/kg. ^2^ Standard error (n = 31 for both diets).

**Table 3:** Effect of the dietary amino acid (AA) concentration from day 77 to 107 of gestation on productive performances in hyperprolific multiparous sows.

Variable	Diets ^1^		SE ^2^	*p*-Value
	Control	High AA		
**AT FARROWING**				
Total born piglets/litter (number)	18.0	19.1	0.543	0.136
Total live-born piglets/litter (number)	15.7	17.1	0.475	0.043
Piglet mortality (%) ^3^	11.6	10.2	1.802	1.000
Litter weight (kg) ^4^	23.1	25.9	0.791	0.017
Litter weight variability (CV)	22.6	23.0	0.984	0.777
Piglet weight (kg)	1.31	1.35	0.037	0.383
**After standardization of litters ^5^**				
Litter weight (kg)	19.8	20.2	0.453	0.556
Piglet weight (kg)	1.52	1.55	0.035	0.572
AT WEANING				
Total weaning piglets/litter (number)	12.4	12.6	0.165	0.786
Litter weight (kg)	67.1	73.0	1.803	0.053
Piglet weight (kg)	5.40	5.79	0.126	0.034
Piglet mortality during lactation (%)	4.47	2.98	1.272	0.786
**SUBSEQUENT PARITY**				
Total born piglets/litter (number)	16.4	18.6	0.500	0.005
Total live-born/litter (number)	15.3	16.9	0.432	0.039
Piglet mortality (%)	6.31	8.67	1.279	0.248

^1^ Control: 6 g standardized ileal digestible lysine -SID Lys-/kg and High AA: 10 g SID Lys/kg. ^2^ Standard error (n = 31 at farrowing and at weaning for both diets and n = 31 and 27 for control- and high AA-diet, respectively, for the subsequent parity). ^3^ Including mummified, stillborn and died during farrowing. ^4^ Only pigs alive born are included. ^5^ The litter size was standardized to 13 piglets per litter.
